# Designing mHealth Apps to Incorporate Evidence-Based Techniques for Prolonging User Engagement

**DOI:** 10.2196/51974

**Published:** 2024-03-26

**Authors:** Rebecca Monachelli, Sharon Watkins Davis, Allison Barnard, Michelle Longmire, John P Docherty, Ingrid Oakley-Girvan

**Affiliations:** 1 Medable Inc Palo Alto, CA United States; 2 Weill Cornell Medical College White Plains, NY United States; 3 The Public Health Institute Oakland, CA United States

**Keywords:** adherence, app design, attrition, mHealth, user engagement, user experience, proof-of-concept

## Abstract

Maintaining user engagement with mobile health (mHealth) apps can be a challenge. Previously, we developed a conceptual model to optimize patient engagement in mHealth apps by incorporating multiple evidence-based methods, including increasing health literacy, enhancing technical competence, and improving feelings about participation in clinical trials. This viewpoint aims to report on a series of exploratory mini-experiments demonstrating the feasibility of testing our previously published engagement conceptual model. We collected data from 6 participants using an app that showed a series of educational videos and obtained additional data via questionnaires to illustrate and pilot the approach. The videos addressed 3 elements shown to relate to engagement in health care app use: increasing health literacy, enhancing technical competence, and improving positive feelings about participation in clinical trials. We measured changes in participants’ knowledge and feelings, collected feedback on the videos and content, made revisions based on this feedback, and conducted participant reassessments. The findings support the feasibility of an iterative approach to creating and refining engagement enhancements in mHealth apps. Systematically identifying the key evidence-based elements intended to be included in an app’s design and then systematically testing the implantation of each element separately until a satisfactory level of positive impact is achieved is feasible and should be incorporated into standard app design. While mHealth apps have shown promise, participants are more likely to drop out than to be retained. This viewpoint highlights the potential for mHealth researchers to test and refine mHealth apps using approaches to better engage users.

## Introduction

Smartphones have a global penetration estimated at 3.9 billion users [1], enabling mobile health (mHealth) apps to reach even low-resource areas and underserved populations [2,3]. mHealth apps have been developed to enable remote participation in clinical trials [4-7] and provide health education, health management, and other uses across the continuum from prevention through active treatment to palliative care [8]. Decentralized clinical trials using mHealth technologies promise faster participant accrual and a higher return on investment than traditional site-based trials [9]. mHealth apps have been shown to reduce inpatient readmission rates and decrease the length of hospital stay [10]. mHealth can increase knowledge and improve confidence and communication with health professionals [11]. However, while participants readily sign up for mHealth education and decentralized clinical trial apps, retention remains a major challenge [12-14]. For mHealth apps to succeed, users must consistently engage with them [15,16].

Engagement in mHealth apps has been conceptualized to include behavior, cognition, and affective components [17]. However, measures of patient engagement are underreported and lack consistency [18,19]. Participants are more likely to drop out than be retained despite app elements such as feedback, reminders, in-app support, gamification, and participant compensation [20-23].

We developed a conceptual model to optimize patient engagement based on different phases of the engagement process [24]. Because digital literacy and anxiety have been shown to be negatively correlated with engagement [25], we established an approach to develop and test the educational components of our conceptual model to enhance app engagement by increasing health literacy, enhancing technical competence, and improving feelings about clinical trials. This Viewpoint aims to report on a series of exploratory mini-experiments, demonstrating the feasibility of testing our engagement conceptual model.

## How We Conducted the Exploratory Mini-Experiments

### Testing Design

We used a product testing approach rather than the traditional research evaluation approach. We used a group of existing product testers who are patients or caregivers working for Medable to rapidly test different iterations of our educational videos. Questionnaires were used both before and after participants viewed the videos, and semistructured interviews were also conducted.

### Data Collection

We developed apps to collect specific data from participants over 1 week’s duration through questionnaires available on their smartphones before and after exposure to videos, as shown in Table 1. The videos were based on a review of the literature defining and studying each of these 3 target areas: health literacy, technical competence, and feelings about participation in clinical trials [24]. Each concept area was tested separately, with questionnaires specific to the educational component.

**Table 1:** Schedule of tasks or questionnaires.

Task or questionnaire	Frequency	Day 1	Day 2	Day 3	Day 4	Day 5	Day 6
Sign up	Once	Task 1	N/A^a^	N/A	N/A	N/A	N/A
Demographics (age, gender, education, housing, race, ethnicity, location, health condition, and notification)	Once	Task 2	N/A	N/A	N/A	N/A	N/A
Technology competence questions (TAM3^b^ CANX,^c^ worries about pressing the wrong button and device preference)	Twice	Task 3^d^	Task 2^e^	N/A	N/A	N/A	N/A
Technology competence video combined with practice questions (video on how to answer questions and practice questions)	Once	N/A	Task 1^d,e^	N/A	N/A	N/A	N/A
Health literacy questions (BRIEF^f^, own health knowledge, clinical trial knowledge, and BMI)	Twice	N/A	N/A	Task 1^d,ed^	Task 2^e^	N/A	N/A
Health literacy video with knowledge check questions (St. Luke’s University Health Network’s video, “Wellness 101 – How to Improve Your Overall Health”; knowledge check questions)	Once	N/A	N/A	N/A	Task 1^d,e^	N/A	N/A
Clinical trials question (temperature scale regarding participant’s feelings about study participation, Figure S1 in Multimedia Appendix 1)	Twice	N/A	N/A	N/A	N/A	Task 1^d,e^	Task 2
Clinical trials video (National Institute of Diabetes and Digestive and Kidney Diseases video: “Why Should I Join a Clinical Trial?”)	Once	N/A	N/A	N/A	N/A	N/A	Task 1^d,e^
Study complete	Once	N/A	N/A	N/A	N/A	N/A	Task 3

^a^N/A: not applicable.

^b^TAM3: Technology Acceptance Model 3.

^c^CANX: Computer Anxiety.

^d^Set up with an automated morning reminder: “You have new tasks available today in the Patient Engagement app!”

^e^Set-up with an automated 8 pm reminder: “You have uncompleted tasks in the Patient Engagement app. Please finish them before midnight!”

^f^BRIEF: Brief Health Literacy Screening Tool.

In addition to collecting data through the questionnaires noted in Table 1, we conducted individual semistructured interviews with each participant at the end of the series of user evaluations using a video conferencing platform. Based on feedback, we revised specific videos and content and then conducted a second round of feedback. In this second round, after all the questions were answered in each section, participants were asked for immediate feedback with the open-ended question, “What did you think of this video?”

### Recruitment

We used a product testing approach rather than a research evaluation approach. We recruited 6 individuals from the Medable Patient Care Network (PCN) who participated in this product development effort from May 2022 through July 2022. The PCN is a group of patients and caregivers who provide insights and user feedback from their perspective for a variety of apps being developed as products at Medable.

### Ethical Considerations

This work was conducted and approved under the the Advarra IRB (Pro00062352). PCN members were paid an hourly rate of approximately US $150 by Medable for their work on behalf of the network in support of Medable product development efforts. Informed consent was obtained from all participants. All data were deidentified.

## Assessment and Interventions

### Health Literacy

The Brief Health Literacy Screening Tool [26] was selected to measure change before and after the health literacy intervention video. This questionnaire has four items that are rated on a 5-point Likert scale from “always” to “never”: (1) How often do you have someone help you read hospital materials? (2) How often do you have problems learning about your medical condition because of difficulty understanding written information? (3) How often do you have a problem understanding what is told to you about your medical condition? (4) How confident are you filling out medical forms by yourself? Two questions were asked in addition to the Brief Health Literacy Screening Tool using a 10-point scale: “How much do you know about your own health,” “How much do you know about clinical trials,” and 1 true or false question: “Do you know your body mass index (BMI)?” (Multimedia Appendix 1).

Before showing a video, a module with a knowledge check portion was included to facilitate pre- and postvideo knowledge comparison. St. Luke’s University Health Network’s video, titled “Wellness 101 – How to Improve Your Overall Health” [27] was chosen for the content area of health literacy. The video provided 5 tips to improve an individual’s overall health. Five knowledge-related questions based on the video were asked. A BMI calculator was included as the final task in this section. Participants were instructed to “Try calculating your BMI on this website” using the CDC BMI calculator [28].

### Technology Competence

We measured internet skills using a section of the Technology Acceptance Model 3 [29]. Statements presented to the participant included “The study website does not scare me at all,” “Working with the study website makes me nervous,” “The study website makes me feel uncomfortable,” and “The study website makes me feel uneasy.” We also asked, “While using the study website, I’m worried that I might press the wrong button and make a mistake that crashes the program” and “I am most comfortable using my (multi-select) iPad/Tablet, Smart Phone (iPhone or Android), Computer, Other.”

The video we used to increase technology competence was created in-house and showed participants examples of how to click on checkboxes, “radio button” response buttons, or move the cursor to a particular spot to answer different types of questions, including multiple choice, multiple selections, and a sliding scale. Participants were then asked to practice answering the same type of questions on their own (Multimedia Appendix 1 for the Technology Competence Questionnaire).

### Clinical Trials

We also asked the question, “When it comes to your feelings about participating in this study, how do you rate your comfort?” using a scale ranging from “0, meaning no distress; totally relaxed” to “100, reflecting the highest anxiety/distress that you have ever felt” (Figure S1 in Multimedia Appendix 1). We showed the video from the National Institute of Diabetes and Digestive and Kidney Diseases, “Why Should I Join a Clinical Trial?” [30].

### Semistructured Interviews

RM and AB conducted 45-minute interviews using Zoom (Zoom Technologies Inc) video conferencing with all 6 study participants using semistructured guides. Open-ended questions explored how participants felt about the process of using the app and how they felt about the questions that were asked through the app. A 5-point Likert scale was used to determine whether they agreed or disagreed with several statements focusing on how useful and informational they felt each of the videos and questionnaires were, for example, “I found the knowledge check questions to be useful” and “I felt less anxious about the idea of participating in a clinical trial after completing the knowledge check.”

### Data Analysis

Given the small sample size and our product testing approach, we used simple descriptive statistics to give us insights into the differences between the “before” and “after” questionnaire results. The semistructured interviews were reviewed for commonalities.

## What We Found

### User Demographics

Users testing the smartphone apps ranged in age from 54 to 69 years. Of the 6 participants, 3 identified as female and 3 as male. Five stated their race as White, 1 selected Black or African American, and none identified as Hispanic. Participants lived in the United States and Europe; 5 owned their homes and 1 rented. The majority (n=5) indicated they had 1 or more health conditions, and 5 had completed at least 4 years of college. All participants indicated they preferred to receive notifications before noon, 4 preferred SMS text message notifications, and 2 preferred email notifications.

### Health Literacy

The mean score and SD for each survey item before and after viewing the instructional video are listed in Table S1 in Multimedia Appendix 1. Lower scores indicated a more positive response for 2 of the questions, specifically, “How confident are you filling out medical forms by yourself?” and “Do you know your Body Mass Index (BMI)?” The mean score for each survey item was slightly more positive after viewing the instructional video, except for the item “How often do you have a problem understanding what is told to you about your medical condition?” However, the SD was greater than the change in mean scores, indicating that it could be due to chance. There were no changes in questionnaire responses before and after watching the instructional video for 3 of the 6 participants, improvement in 2 questions and a decline in 1 question for 1 participant, improvement in 4 questions and no change in 3 questions for 1 participant, and improvement in knowledge of their overall health and a decline in the clinical trial knowledge item for 1 participant.

Participants’ feedback from the semistructured interviews revealed negative feelings toward the video “Wellness 101 – How to Improve Your Overall Health.” One participant described the video as “juvenile,” while another noted concerns that some participants might object to the health video if they already smoke or have a high BMI. All participants agreed or strongly agreed to the question, “I found the knowledge check questions to be useful.” In total, 4 of the 6 participants neither agreed nor disagreed with the statement, “I felt less anxious about the idea of participating in a clinical trial after completing the knowledge check.” Participants liked the alternative health literacy video, “5 Ways to Make the Most of Your Doctor Visit” [31].

### Technology Competence

The mean scores for all items were more positive after viewing the video. The scores for 3 participants improved after watching the instructional video but declined for 2 participants. There was no change for 1 participant (Table S1 in Multimedia Appendix 1).

Participants (N=6) shared positive feedback about the video showing how to answer and practice questions. When asked, “Watching somebody else demonstrate how to answer questions made me feel like I knew what was expected of me in the study,” participants answered, “agree” (2 participants) and “strongly agree” (4 participants). Additionally, participants answered “strongly agree” (4 participants) to the survey, “Practicing answering questions on my own made me feel less anxious about participating in the study.” Some participants felt the questions in this section were redundant and thought all the questions could be combined into 1 question. In addition, participants thought the questions felt negative with the emphasis on the terms “anxious” and “nervous” and suggested changing the questions to make them seem more positive. One participant suggested making the technology anxiety section optional for those who feel more comfortable using the study website.

### Clinical Trials

Mean scores were slightly higher prior to viewing the instructional video compared with after the video. One participant improved by 10 points, 1 decreased by 10 points, and the other 4 stayed the same.

Participants had positive things to say about the video “Why Should I Join a Clinical Trial?” [30] When asked, “I found the video to be useful,” 2 participants answered “agree” and 4 answered “strongly agree.”

### Additional Overall User Feedback

The participants had several specific suggestions. One participant suggested making sure that the videos were clearly specific to diseases or therapy areas in the trial and gave specific information on the trial structure. Two participants suggested including additional content on participant safety. We did an ad hoc assessment of this suggestion and sent the National Cancer Institute’s “Patient Safety in Clinical Trials” video for feedback [32]. The majority (n=5) participants liked the video. The National Institute of Mental Health’s video, “What are the risks and benefits of participating in clinical research?” was also considered [33]. Most (n=4) participants liked the video and 2 did not. Some participants thought the video was juvenile and better for children or young adults. Others also thought the video did not explain concepts such as placebos well. In response to this feedback, the following resources from the National Institute on Aging were added: “What are Clinical Trials and Studies?” [34] and “Clinical Research Benefits, Risks and Safety” [35], after which we sought a second round of feedback. Most participants (n=5) liked the additional resources.

## Discussion

### Key Lessons

Maintaining continuous and complete use of mHealth apps has remained a persistent problem that has not yielded even sophisticated solutions such as timed and individualized user messaging. A newer and evolving understanding of the foundational importance of user engagement with mHealth suggests that this problem comes from a lack of appreciation by mHealth app designers of the complex and multicomponent structures behind user engagement. We have built on prior knowledge and work to develop a model of engagement that accounts for the complexity of engagement [24].

This viewpoint was an exploratory study to determine the feasibility of this approach and to guide the refinement of this interactive test strategy. We learned several key lessons: (1) Specificity—the participants endorsed the recommendation that the interventions should be specific to the educational needs of the target of the mHealth app. The most positive feedback was given to the video we developed de novo to teach participants the technical competence required to correctly and effectively use the app to report their evaluations. (2) Attention to inadvertent adverse affective variables—the participants noted the importance of avoiding or rephrasing medical terms that could be seen as demeaning by some participants (eg, obesity, age, and infirmity). (3) Individualization—the participants clearly reflected different levels of need for improving their technical competence and health literacy. Our results indicate the potential importance of personalization in health app design addressing individuals’ levels of need and cultural and personal sensitivities. For example, a way to allow more individualization is to allow users to potentially opt out of certain learning features if they do not think they need them.

### Comparison With Prior Work

Other studies have assessed how to adjust apps to increase engagement. One study found positive effects on adherence from personalization or tailoring of the app content to users’ needs, push notification reminders, user-friendly design, and personal support along with digital intervention [36]. However, the high dropout rate in app usage remains a major challenge [37]. A recent literature review found that despite factors such as appropriate reminders and feedback, app participants were more likely to drop out than be retained [20]. App literacy skills have been identified as a major factor in the uptake and engagement of smartphone apps [38]. Although we identified studies recommending web-based interventions to increase health literacy and technical skills [39,40], we have not found other studies testing approaches to increase those skills.

### Limitations and Strengths

Our sample size was limited to 6 participants, 5 of whom were highly educated. We were unable to use any statistical significance measures because of the small sample size or draw conclusions that would apply to a larger population. Our highly educated sample is a limitation because these individuals may have better digital literacy than the general population. The main goal of our series of mini-experiments was to assess the feasibility of our approach and see whether we could retain the interest of the participants and obtain useful feedback on the interventions, which was successful. This type of testing is intended to evaluate tailored iterations of the app after gathering rapid participant feedback, with the ultimate goal of developing an app that will engage users. The next phase of our work will be to undertake the systematic testing of each component of this model in a larger and more diverse sample. We will then be able to refine the interventional enhancements for those components for a broader population. Ultimately, the functional use of this approach requires much larger, population-specific samples.

### Conclusions

To fulfill the promise of mHealth apps to improve health outcomes, apps need to be improved so they reduce participant attrition. Health care apps do not work for people who do not use them. To date, app feedback, notifications and reminders, in-app support, gamification, and participant compensation have not been consistently successful in eliminating participant dropout. This study highlights the potential to develop and refine mHealth apps using evidence-based interventions derived from a broad range of behavioral and social science to increase engagement as a way of improving participant retention. This viewpoint highlights the potential for mHealth researchers to test and refine mHealth apps using approaches to better engage users. The preliminary experience reported in this viewpoint supports the feasibility of this iterative approach to create and refine engagement interventional enhancements for each element of the multidimensional, multicomponent theory of the engagement process.

## Appendix

Multimedia Appendix 1 Study questionnaires and responses.

## Abbreviations

mHealth: mobile health
PCN: Patient Care Network

## Data Availability

The data sets generated and analyzed during this study are not publicly available due to the data release requirements of the Patient Care Network and the intellectual property associated with this work but are available from the corresponding author on reasonable request.
