# Supplementary material for: Designing mHealth Apps to Incorporate Evidence-Based Techniques for Prolonging User Engagement
Source: Interact J Med Res. 2024 Mar 26;13:e51974. doi: 10.2196/51974 (PMC11005439; doi:10.2196/51974)
Supplement: Multimedia Appendix 1 [file ijmr_v13i1e51974_app1.docx]

**A.** **Health Literacy Questionnaire**

1. How often do you have someone help you read hospital materials?
2. How often do you have problems learning about your medical condition because of difficulty understanding written information?
3. How often do you have a problem understanding what is told to you about your medical condition?
4. How confident are you filling out medical forms by yourself?
   - 1. 1 - Always
     2. 2 - Often
     3. 3 - Sometimes
     4. 4 - Occasionally
     5. 5 - Never
5. How much do you know about your own health?
   1. Scale from 1-10
   2. Anchor points- Nothing to Everything
6. How much do you know about clinical trials?
   1. Scale from 1-10
   2. Anchor points- Nothing to Everything
7. Do you know your Body Mass Index (BMI)?
   1. (YES OR NO)

**B. Technology Competence Questionnaire**

1. *CANX1:* The study website does not scare me at all.
2. *CANX2:* Working with the study website makes me nervous.
3. *CANX3:* The study website makes me feel uncomfortable.
4. *CANX4:* The study website makes me feel uneasy.
   - 1. 1 - Strongly Disagree
     2. 2 - Moderately Disagree
     3. 3 - Somewhat disagree
     4. 4 - Neutral (Neither disagree nor agree)
     5. 5 - Somewhat agree
     6. 6 - Moderately agree
     7. 7 - Strongly agree
5. While using the study website, I’m worried that I might press the wrong button and make a mistake that crashes the program.
   1. Sliding Scale 1-10
   2. Add anchor points:
      1. Not worried at all
      2. Extremely worried
6. I am most comfortable using my (multi-select):
   1. iPad/Tablet
   2. Smartphone (iPhone or Android)
   3. Computer
   4. Other

**Figure S1.** Feelings about clinical trials question response scale.


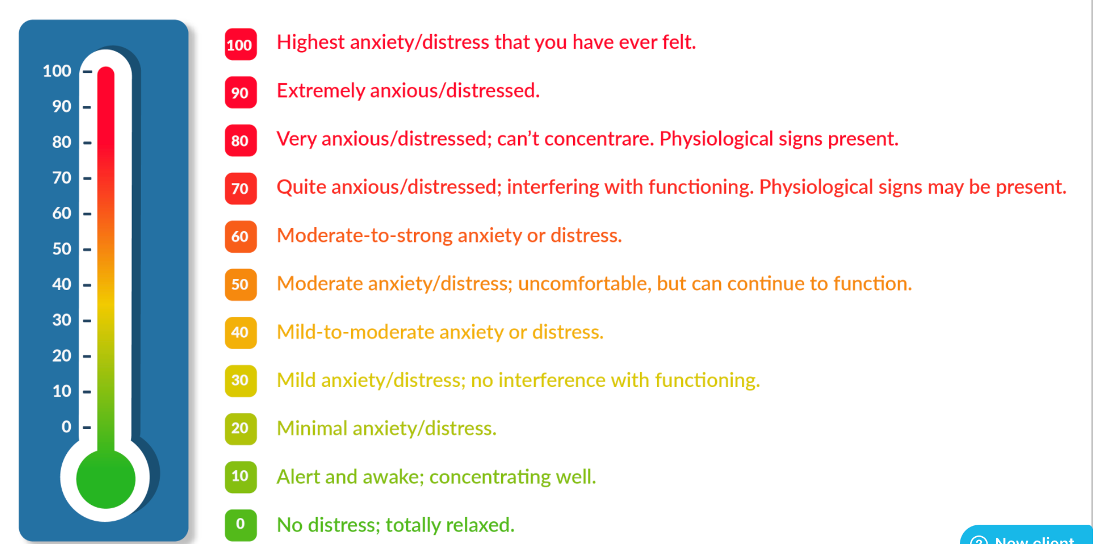


**Table S1.** Questionnaire responses before and after instructional videos (mean score and standard deviation for N=6 participants.^a^

| **Question** | **Before**  **Mean score**  **(± SD)** | **After**  **Mean score**  **(± SD)** |
| --- | --- | --- |
| Health Literacy  How often do you have someone help you read hospital materials?  How often do you have problems learning about your medical condition because of difficulty understanding written information?  How often do you have a problem understanding what is told to you about your medical condition?  How confident are you filling out medical forms by yourself?  How much do you know about your own health?  How much do you know about clinical trials?  Do you know your Body Mass Index (BMI)? | 4.36 (± 1.03)  3.91 (± 1.22)  **4.30** (± 0.81)  1.82 (± 1.17)  8.27 (± 1.10)  7.36 (± 1.91)  1.33 (± 0.52) | **4.45** (± 1.04)  **4.18** (± 1.08)  4.25 (± 0.87)  **1.55** (± 0.93)  **8.45** (± 1.04)  **7.45** (± 1.92)  **1.18** (± 0.40) |
| Technology Competence  The study website does not scare me at all.  Working with the study website makes me nervous.  The study website makes me feel uncomfortable.  The study website makes me feel uneasy.  While using the study website, I’m worried that I might press the wrong button and make a mistake that crashes the program. | 6.00 (± 1.34)  1.91 (± 1.38)  1.91 (± 1.38)  2.00 (± 1.34)  2.36 (± 2.20) | **5.91** (± 1.30)  **2.00** (± 1.34)  **2.00** (± 1.34)  **2.09** (± 1.30)  **2.64** (± 2.20) |
| Clinical Trials  When it comes to your feelings about participating in this study, how do you rate your comfort? | **6.66** (± 7.78) | 7.27 (± 7.78) |

^a^ Scores indicating the more positive response are bolded.
